# Pharmacokinetic modeling of sulfamethoxazole-trimethoprim and sulfadiazine-trimethoprim combinations in broilers

**DOI:** 10.1016/j.psj.2024.104200

**Published:** 2024-08-08

**Authors:** Marine Boulanger, Jean-François Taillandier, Jérôme Henri, Mathias Devreese, Siegrid De Baere, Aude A. Ferran, Alexis Viel

**Affiliations:** ⁎ Therapeutic Innovations and Resistance (INTHERES), Université de Toulouse INRAE ENVT, Toulouse 31076, France; † Fougères Laboratory, French Agency for Food, Environmental and Occupational Health & Safety (ANSES), 10B rue Claude Bourgelat, Fougères 35306, France; ‡ Department of Pathobiology, Laboratory of Pharmacology and Toxicology, Pharmacology and Zoological Medicine, Faculty of Veterinary Medicine, Ghent University, 9820, Merelbeke, Belgium

**Keywords:** sulfonamide, trimethoprim, combination, dosage regimen, population pharmacokinetic modeling

## Abstract

Sulfonamides (**S**) are old bacteriostatic antibiotics which are widely prescribed in combination with trimethoprim (**TMP**) for the treatment of various diseases in food-producing animals such as poultry. Nowadays, the 1:5 dose ratio of TMP/S used in broilers is a direct transposition of the ratio determined in Human decades ago for TMP/sulfamethoxazole (**SMX**), aiming to obtain a supposed synergistic plasma concentration ratio of 1:19. However, major pharmacokinetics **(PK)** differences exist according to the sulfonamide used in the combination. Here, we generated new PK data in broilers after a cross-over design with IV and the oral administration of 2 major sulfonamides, sulfadiazine **(SDZ)** and SMX, in combination with TMP, and analyzed the data via a population pharmacokinetic **(popPK)** modeling approach. Results showed that TMP has a greater plasma to tissue distribution than both sulfonamides with a higher volume of distribution (0.51 L/kg for SDZ, 0.62 L/kg for SMX and 3.14 L/kg for TMP). SMX has the highest elimination half-life (2.83 h) followed by SDZ and TMP (2.01 h and 1.49 h, respectively). The oral bioavailability of the 3 molecules was approximately 100%. Bodyweight could explain some of the inter-individual variability in the volume of distribution of SDZ and SMX and the clearance of SDZ and TMP, as heavier broilers have higher typical values. Monte Carlo simulations of a large virtual broiler population (n = 1,000) showed that the targeted plasma ratio of TMP:S of 1:19 was rarely or never reached at the individual level for both combinations at the marketed doses and greatly varies over time and between individuals, questioning the relevance of the 1:5 dose ratio for current formulations of TMP/S.

## INTRODUCTION

In the 2022 report of the European Medicine Agency's (**EMA**), sulfonamides (**S**) were the third most sold antibiotics in food-producing animals across 31 European countries (European Medicines Agency, 2022). Sulfonamides are a large family of synthetic, bacteriostatic antifolate molecules that inhibit the biosynthesis of important tetrahydrofolate coenzymes. All sulfonamides share the same antimicrobial action by competing with *p*-aminobenzoic acid to inhibit the dihydropteroate synthase. Their antimicrobial activity is very often potentiated by diaminopyrimidines, such as trimethoprim (**TMP**), which inhibit the reduction of dihydrofolic acid via competitive antagonism with dihydrofolate reductase. Sequential inhibition of the same metabolic pathway makes the combination bactericidal and synergistic against gram-positive and gram-negative bacteria, protozoa, and coccidia (Bushby, 1980; Masters et al., 2003). Both antibiotics were recently classified by the EMA as category D (“Prudence”), which promotes their use by veterinarians as first-line treatments compared to other families of antibiotics that are considered more critical to human health and are classified in class A (e.g. carbapenems) to C (e.g. macrolides) (Thompson et al., 2019; European Medicines Agency, 2020).

In poultry production, veterinary drugs are mainly administered as a flock treatment through water drinking or feed medication (Vermeulen et al., 2002). The TMP:S dose ratio in almost all veterinary formulations on the market is 1:5. This ratio was historically developed for the combination of TMP/sulfamethoxazole **(SMX)** in human medicine as it allows an *in vivo* plasma concentration ratio close to 1:19 to be obtained, which is considered to be the most synergistic ratio against a wide range of human pathogens. This ratio of plasma concentrations of TMP:SMX is relatively constant over time in humans as both molecules have approximately the same elimination half-life **(T_1/2β_)** (Thompson et al., 2019). However, in food-producing animals such as broilers, different sulfonamide drugs with different chemical structure and pharmacokinetics **(PK)** are used, which challenges the direct translation of doses administered to humans to food-producing animals and the relevance of this constant 1:19 ratio for TMP:S in plasma (Bushby, 1980). For example, Baert et al. (2003) showed that after an oral bolus administration of the combination TMP/sulfadiazine (**SDZ**) at a dose ratio of 1:5, a plasma concentration ratio of 1:25 was only reached for 1 to 4 h in broilers. Approximately 6 h after administration, the ratio was 1:70-90 (Baert et al., 2003). However, even if this "optimal" plasma ratio of 1:19 is not observed, this does not mean that the efficacy and the synergistic effect are suddenly lost with other ratios. In fact, the optimal ratio should vary greatly from strain to strain, as it would be equal to the ratio of the minimum inhibitory concentration (**MIC**) of the drugs when acting alone (Bushby, 1980; Salter, 1982). The only available MIC distributions used in veterinary medicine are determined by EUCAST and only for the combination TMP/SMX at the ratio 1:19. This *in vitro* ratio does not seem to be representative of the *in vivo* ratios of plasma concentrations obtained in broilers, making it difficult to compare the susceptibility between commonly found pathogens in broilers and the expected efficacy of current TMP/S dosing regimens.

To date, there is a paucity of data in the literature on the combination of TMP/S in broilers, mainly relating to SMX and SDZ. In addition to the lack of information, the data have been analyzed using noncompartmental analysis **(NCA)**. A more informative and optimal approach than NCA would be the use of nonlinear mixed effect **(NLME)** modeling, which allows the effect of inter-individual variability (and residual variability) to be identified, explored and quantified based on the individual PK data. Population PK **(PopPK)** modeling can serve a wide range of purposes, including the optimization of existing dosing regimens and is a useful approach for analyzing sparse sampling data when extensive blood sampling is difficult for some species such as poultry. PopPK modeling consists of generating a PK profile for each animal and then developing a model that fits all of the individual profiles (Bon et al., 2018). It also takes into account and quantifies the inter-individual variability, the residual variability and the effect of the several main factors called covariates (bodyweight, age, sex…). These estimated variabilities and covariates can then be used to predict the efficacy of a treatment with TMP/SDZ and TMP/SMX at the flock level or to propose optimized dosing regimens that could achieve 80-90% efficacy in a flock.

Another limitation of the available PK data is the fact that most recent studies have either focused on the oral route only, thus providing only apparent PK parameters or have used analytical methods with relatively high limits of quantification **(LOQ)** of 1 µg/mL for SMX and 0.2 µg/mL for TMP (Petritz et al., 2023). The elimination half-life of SDZ is reported to be approximately 3 h (Löscher et al., 1990; Baert et al., 2003), whereas the mean T_1/2β_ values for SMX ranged from 3.6 h (Petritz et al., 2023) to 8.25 h (Queralt and Castells, 1985). The T_1/2β_ of TMP is reported to vary between 1 h and 3.9 h (Queralt and Castells, 1985; Löscher et al., 1990; Baert et al., 2003; Petritz et al., 2023).

Drug exposure *in vivo* should be optimal to avoid suboptimal concentrations of antibiotics which are known to trigger the selection of resistance in pathogenic bacteria (Lees et al., 2019) but also in commensal bacteria that could then spread towards the environment (Szmolka and Nagy, 2013; Dréano et al., 2022). Therefore, it is crucial to optimize the dosing regimens (dosage and frequency) of currently available drugs for poultry not only to achieve a maximal antibacterial effect but also to reduce the potential emergence of resistance. The aim of this study is to re-examine the kinetics of SDZ or SMX in combination with TMP in broilers using population PK modeling and to estimate the distribution of the TMP:S ratio in broilers over time following administration at the population (or flock) level. This will guide drug dosing regimens tailored to the target species.

## MATERIALS AND METHODS

### Animals

The animal study included 39 healthy broilers (breed: Ross 308) aged 3 to 4 wk old at the start of the study and purchased from 2 local breeders in Brittany (France). Two independent cross-over studies (for intravenous [**IV**] and oral routes) were conducted with 19 broilers (13 males and 6 females) and 20 broilers (9 males and 11 females) for the combinations TMP/SDZ and TMP/SMX, respectively. The broilers were housed in groups of 5 in a pen and had access to fresh antibiotic-free food and water *ad libitum* throughout the experiment. Their environment was climate-controlled with a 12-h light:dark cycle and enriched with perches, ropes and sawdust bedding. Animals were acclimatized for 1 wk before being enrolled in the study. The welfare and health of the broilers were visually monitored at least once daily throughout the study. At the end of the crossover, all animals were killed by electronarcosis followed by exsanguination. The study was approved by the French Committee on Animal Research and Ethics (APAFIS #39536- 2022110811424657 v4).

### Drugs and Chemical Reagents

Diatrim (Eurovet Animal Health, Bladel, The Netherlands; 200 mg SDZ + 40 mg TMP per mL) and Adjusol (Virbac, Carros, France; 83.35 mg SDZ + 16.65 mg TMP per mL) containing SDZ and TMP were used for IV injection and oral administration, respectively. Lidoprim S® (Prodivet pharmaceutical sa/nv, Eynatten, Belgium; 200 mg SMX + 40 mg TMP per mL) and T.S SOL (Dopharma Research, Raamsdonksveer, The Netherlands; 100 mg SMX + 20 mg TMP per mL) containing SMX and TMP were used for IV injection and oral administration, respectively. All drugs contained TMP:S at a concentration ratio of 1:5 and were half-diluted in saline solution or drinking water prior to IV injection or oral administration, respectively.

For the analytical experiments, analytical standards of SDZ, SDZ-d4, SMX, SMX-d4, TMP and TMP-d9 were obtained from Sigma Aldrich (Overijse, Belgium), TRC Canada (North York, ON, Canada), Sigma Aldrich, Cayman Chemicals (Ann Arbor, MI, USA), Sigma Aldrich and Merck (Darmstadt, Germany), respectively. Purity of the analytical standards was 99.7%, 96.85%, 99.7%, 99.7%, 99.5% and 99.8%, respectively, and was accounted for in the analyses. Ethylacetate and acetic acid were obtained from Merck whereas acetonitrile was obtained from Fisher Chemicals (Belgium). UPLC grade water was produced freshly using a Milli-Q system (Merck, Overijse, Belgium).

### Experimental Study and Blood Sample Collection

Twenty-four hours prior to dosing, broilers were weighed and randomly assigned to the IV or oral group for the first period of the cross-over design (9 or 10 chickens for each group). The mean weight of each group was used to prepare the solutions of TMP/SDZ or TMP/SMX and the broilers were not fastened before any of the administrations (IV or oral). During the experimental study, the weight of the broilers ranged from 1.07 kg to 2.98 kg and from 1.86 kg to 2.65 kg for the combinations TMP/SDZ and TMP/SMX, respectively. For the IV injection, they were manually restrained and a spot catheter (Introcan W-certo 24G B.Braun) was placed in their right or left wing vein. The catheter was briefly flushed with physiological saline solution before slowly injecting 0.16 mL/kg of the solutions containing TMP/SDZ or TMP/SMX (equivalent to 32 mg/kg of SDZ or SMX + 6.4 mg/kg of TMP) as a single dose. After the injection, the intravenous catheter was again flushed with physiological saline before removal. For the oral administration by gavage, broilers were manually restrained and received 0.35 mL/kg of the solutions containing TMP/SDZ (equivalent to 29.2 mg/kg of SDZ + 5.8 mg/kg of TMP) or 0.37 mL/kg of TMP/SMX (equivalent to 37.5 mg/kg of SMX + 7.5 mg/kg of TMP) through a feeding tube inserted in their crop. After a washout period of 5 d following the last sampling, all groups were reversed: the IV group received the same TMP/S combination by oral administration and the oral group received the same TMP/S combination by IV injection.

Blood samples (0.2–0.6 mL) were collected in heparinized tubes alternatively on the right and left leg, or through the occipital sinus. A sparse blood collection design allowed sampling at 0.083h (IV only), 0.25, 0.5, 0.75, 1, 1.5, 2, 3, 4, 6, 8, 10, 12, 24 and 32 h after drug administration for both combinations. Each broiler was sampled between 7 and 8 times over 32h corresponding to a total of 14 to 16 sampling times throughout the entire experiments. Samples were then centrifuged within 30 min of collection during 10 min, 3,000 x *g* at 5°C and plasma were stored at −20°C until drug quantification.

### Drug Concentrations Analysis

Sample preparation consisted of pipetting 100 µL sample of plasma in an Eppendorf cup, to which 25 µL of an internal standard solution (= SMX-d4 or SDZ-d4 at 25 µg/mL and TMP-d9 at 10 µg/mL, used respectively as internal standards for SMX/SDZ and TMP measurement) and 100 µL of acetonitrile (as the extraction solvent) were added successively. The sample was then homogenized at 2,500 rpm for 1 min and subsequently centrifuged at 13,000 rpm for 10 min at 4°C. The supernatant was transferred in a 15 mL tube with 1.5 mL ethylacetate added and swayed on a roller bank for 15 min. Next, the upper part was transferred in a different 15 mL glass centrifuge tube and the solvent was evaporated under a gentle nitrogen flow at 40°C. The extract was redissolved in 125 µL of 0.1 % (v/v) acetic acid in Milli-Q water and transferred in a glass conical autosampler vial. A 5-µl aliquot was injected on the UPLC-MS/MS system.

The UPLC-MS/MS instrument consisted of a Quattro Premier XE instrument (Waters, Antwerp, Belgium). For chromatographic separation, an Acquity UPLC BEH C18 (2.1 × 50 mm, 1.7 µm) column, in combination with a VanGuard precolumn of the same type (2.1 × 5 mm) was used. Mobile phase A comprised 0.1% (v/v) aced acid in water and mobile phase B was acetonitrile. A gradient elution program was used: 0 to 3.0 min, 90%A/10%B, 3.0 to 5.5 min, to 5.0% A/95.0% B, 5.5 to 5.7 min, to 90.0% A/10.0% B, 5.7 to 7.5 min, 10.0% A/90.0. Flow rate was maintained at a constant rate of 0.45 mL/min. The sample compartment was cooled at 8°C, while the UPLC column was maintained at a temperature of 30°C. The flow was sent by means of a divert valve from 1.1 min to 2.0 min and from 3.0 to 4.3 min, i.e. the time window in which the analytes elute from the UPLC column, to the triple quadrupole mass spectrometer. Quantification of the compounds was achieved by means of component-specific MRM (Multiple Reaction Monitoring) transitions. Quantification was based on the ratio of the analyte peak area to the peak area of its corresponding internal standard. The following mass to charge ion (***m/z***) transitions were used: SMX: 253.87 (precursor ion) > 156.01 and 92.00 (product ions), collision energy (**CE**): 15 V; SMX-d4: 258.01 > 160.21, 96.16, CE 15 V; TMP: 291.13 > 230.14, 123.06, CE 25 V; TMP-d9: 300.14 > 123.08, 243.33, CE 25 V.

Method validation consisted of using blank broiler chicken plasma samples. The matrix-matched calibration curve ranged from 4 - 4,000 ng/mL levels (including 4, 10, 20, 40, 100, 200, 400, 1,000, 2,000 and 4,000 ng/mL) for TMP and for SMX/SDZ the calibration curve ranged from 20 to 100,000 ng/mL (including 20, 50, 100, 200, 500, 1,000, 2,000, 5,000, 10,000, 20,000, 25,000, 30,000, 50,000, 60,000, 80,000, 100,000 ng/mL).

For the sulfonamides, within-run accuracy and precision were evaluated on n = 6 replicates at the 50, 500, 5,000, and 50,000 ng/mL levels, prepared in the same way as the calibrator samples. Between-run accuracy and precision were performed on 3 different days with n = 6 replicates on each day. For TMP, the same approach was used with replicates at 10, 20, 100 and 1,000 ng/mL. The limit of quantification was 4 and 20 ng/mL for both TMP and SDZ/SMX, respectively.

### Population PK Modeling

Plasma concentration data of SMX, SDZ and TMP were analyzed using a nonlinear mixed effect model with Monolix (Lixoft, version 2023R1) and pop PK adjustments were realized according to the SAEM algorithm, taking into account below the limit of quantification (**BLQ**) data as left-censored. All data were analyzed simultaneously, that is, a separate model was developed for each drug but the parameters for the TMP model were shared between the 2 experimental designs. Several structural models (i.e. 1- and 2-compartments) were tested and selected according to their Corrected Bayesian Information Criteria **(BICc)** and graphical evaluations of the data (plots of the population weighted residuals **(PWRES)**, of the individual weighted residuals **(IWRES),** of the normalized prediction distribution errors [**NPDE**]). The primary population pharmacokinetic parameters determined were the volume of distribution **(Vd)**, the clearance **(CL)**, the absolute bioavailability **(F)** and the rate of absorption **(ka)** for the 3 molecules. The elimination half-life of each molecule and the area under the curve from time 0 to infinity **(AUC_0-∞_)** were also calculated as secondary parameters. The population parameters were estimated according to a lognormal distribution except for F which was estimated according to logit normal distribution which allowed to restrict the distribution limit between 0 and 1.

The mixed-effect model combines (1) the fixed effects which are population parameters that are assumed to be the same for all individuals in the studied population, determined with a structural model and (2) the random effects which are subject-specific random variables, determined with the random model including the inter-individual variability **(IIV)**, the covariate model that could explain part of the IIV and the residual (unexplained) variability (e.g. analytical variability, dosing or sampling errors, model misspecification…) (Bon et al., 2018).

Covariates are important to highlight potential relationships between estimated popPK parameters and biological characteristics of the subjects (e.g. age, breed, sex) which may ultimately explain some of the variability. In this model, “body weight” **(BW)** and “sex” were the 2 biological covariates implemented for SDZ, SMX and TMP.

The full model is described in equation (1):

(1) $$\text{Log}\left(H_{i}\right)=\text{log}\left(H_{\text{pop}}\right)+\beta_{\left[\text{sex}=M\right]}+\beta_{\text{BW}}\times \text{log}\left(\text{BW}/1.91\right)+\eta_{\text{Hi}}$$

where H_i_ is the value of the structural parameter H (Cl, ka and Vd) for a given individual i, H_pop_ is the value estimated for the population, β_[sex ==M]_ is the value for the relationship with the covariate sex male (M) (female (F) being the reference status), BW is the bodyweight of the individual i, β_BW_ is the value for the relationship with the bodyweight normalized to its median value (1.91 kg) and η_Hi_ is the random effect for the parameter H of the individual i. Beta values were kept in the model only if they were significatively different from zero.

Another interest of the population PK approach is to take into account correlations between random-effects. It was important to include correlations in the model and then in the simulation of a new cohort of individuals which avoided unrealistic simulations with variability that could have been larger than the reality. Correlations between random-effects were estimated using a full variance-covariance matrix. They were kept in the virtual simulations (see below) when they were higher than 0.2. Finally, the residual error was best described by a proportional error model for SDZ, SMX and TMP.

An internal validation was carried out based on the predicted-corrected visual predictive check **(pcVPCs)** (Bergstrand et al., 2011).

### Simulations

The final pop PK model, including IIV, covariate relationships and correlations (correlation values >0.2) between random effects was then exported from Monolix to Simulx (Lixoft, version 2023R1) to generate a large virtual population of broilers by Monte Carlo simulation (n = 1,000). First, an external validation was performed to assess the predictive ability of the final model, using mean data extracted with WebPlotDigitizer (version 4.7) from Baert et al. (2003) and Löscher et al. (1990) studies (Cheng et al., 2021). Simulations of the total plasma drug concentration over time were generated by considering the tested doses (33.34 mg/kg BW of SDZ + 6.367 mg/kg BW of TMP for Baert et al. (2003) study and 100 mg/kg of SDZ + 20 mg/kg of TMP for the study by Löscher et al. (1990) and the mean animal weight used in these studies (1.9 ± 0.1 kg for the study by Baert et al. (2003) because their broilers had weights between 1.8-2.0 kg and 0.825 ± 0.2 kg for the study by Löscher et al. (1990) because their broilers were between 17 and 22 d). The 90% prediction interval of these simulations was plotted against the extracted data from the literature to assess the predictive availability of the popPK model.

The evolution of the ratio of (free) concentrations of TMP/SDZ and TMP/SMX over time was then simulated at the population level (n = 1,000) for oral administration of registered doses corresponding to 25 mg/kg of SDZ + 5 mg/kg of TMP and 37.5 mg/kg of SMX + 7.5 mg/kg of TMP for Adjusol and T.S SOL respectively. Free concentrations and then, ratios of free concentrations were obtained by correcting the total concentrations with the protein binding values for SDZ (80%) and TMP (77%) obtained from the summary of product characteristics of Adjusol (Summary of product characteristics of Adjusol, 2024) assuming a linearity of these values over the concentration range. For SMX, in the absence of a value for chicken, protein binding was set at a value of 62%, as found in cows and sheep (Nielsen and Rasmussen, 1977; De Backer et al., 1981).

## RESULTS

### Single IV and Oral Administration of TMP/SDZ and TMP/SMX

**Figure 1:**
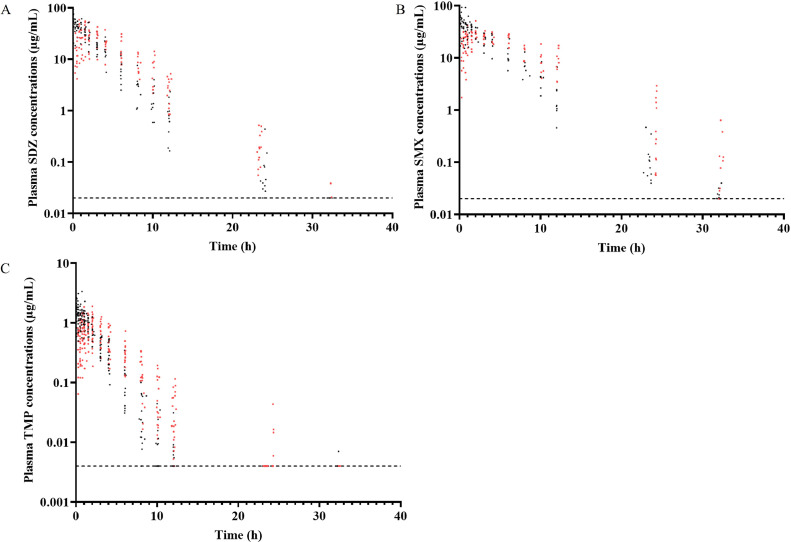
Scatter plot of the plasma concentration of (A) SDZ, (B) SMX and (C) TMP versus time following a single IV (black) and oral (red) administration (32 mg/kg of SDZ + 6.4 mg/kg of TMP for the IV administration and 29.2 mg/kg of SDZ + 5.8 mg/kg of TMP or 37.5 mg/kg of SMX + 7.5 mg/kg of TMP for the oral administration). All broilers (n = 19 for SDZ, n = 20 for SMX and n = 39 for TMP) are represented and each dot corresponds to 1 sampling. The limit of quantification (LOQ) for both sulfonamides is 0.02 µg/mL and 0.004 µg/mL for TMP and is represented with a black dashed line. Data below the LOQ were put at the LOQ value.

The broilers showed no signs of distress or loss of appetite during the 2 wk of the cross-over. However, following the IV injection of TMP/SDZ or TMP/SMX, almost all broilers immediately exhibited physiological reactions such as general weakness, watery eyes and difficulty swallowing, which disappeared spontaneously in less than 1 or 2 min. Plasma concentrations versus time after dosing are shown in Figure 1 for SDZ, SMX and TMP. Drug concentrations were above the LOQ up to 12 h to 24 h after the administration of TMP, until 24 h after the administration of SDZ and 24 to 32 h after the administration of SMX. Data for which unexpected events occurred during sampling (first time point following a perivenous risk) or obvious inconsistencies in drug concentrations were considered as outliers and therefore excluded (n = 132 outliers over 1,142 data). Otherwise, all data were included for the analysis. All PK data collected and used has been published and is available (https://doi.org/10.57745/D0R0Q5).

### Population PK Analysis

**Figure 2:**
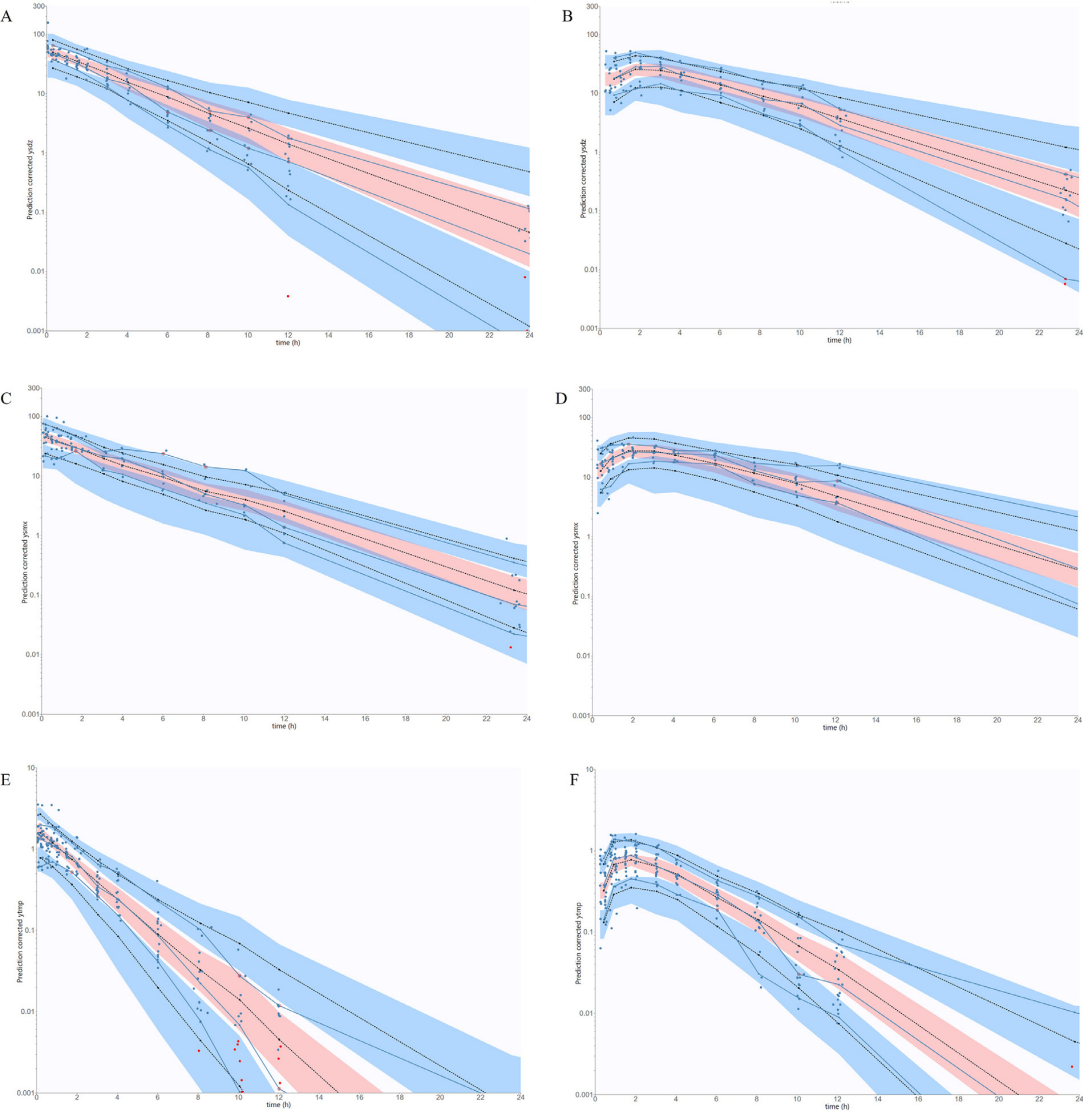
Predicted-corrected VPCs of SDZ (at the top), SMX (in the middle) and TMP (at the bottom) following (A-C-E) an IV or (B-D-F) an oral administration. Data after 24 h are not represented. Experimental data are represented in blue dots, censured data are represented in red dots, the empirical percentile is represented in blue and the predictive percentile is represented in black dashed lines. The 10% and 90% interval are represented by the upper and lower blue areas and the median is represented by the red area.

A 1-compartment structural model was found to best fit the 3 antibiotics according to the BICc comparison and the goodness of fits plots (PWRES, IWRES, NPDE) (see supplementary data Figure 1). Values of the estimated population parameters (V, CL, ka), the standard deviation of the random effect and the error model parameter for SDZ, SMX and TMP are presented in Table 1. Details of the correlation values obtained thanks to the full variance-covariance matrix can be found in the supplementary data (Supplementary Data Table 1). The clearance of SMX and SDZ were found to be similar (around 0.15 L/h/kg) and 10 times lower than the clearance of TMP (1.53 L/h/kg). Heavier broilers had a higher clearance of SDZ and TMP, indicating a significant effect of bodyweight on this population parameter. The volume of distribution of TMP was found to be 3.14 L/kg and about 6 times higher in comparison to the Vd of SMX and SDZ (0.62 L/kg and 0.51 L/kg, respectively). Again, the bodyweight was found to have a significant positive influence as heavier broilers showed higher SDZ and SMX volumes of distribution. The secondary parameters such as AUC from time 0 to infinity and half-lives were calculated for each molecule following the 2 routes of administration and are summarized in Table 2. The oral parameters (ka) were found to be the same between TMP and SDZ (0.59 h^−1^), with a slight exception for SDZ (0.71 h^−1^) (see Table 1). The predicted-corrected VPCs for the 3 models (one for each molecule) and for each route of administration are presented in Figure 2. Overall, the final models for each drug gave good predictions of observed data with only minor misspecifications and were thus considered as validated. For the external validation of SDZ and TMP models, mean data from Löscher and Baert studies were included within the defined 5% to 95% interval, strengthening the good predictive ability of both models (Löscher et al., 1990; Baert et al., 2003).

**Table 1:** Values of estimated population parameters, standard deviation of the random effects and residual error variability for each model using nonlinear mixed effect (NLME) following a single IV and an oral administration of TMP/SDZ or TMP/SMX to broilers.

Fixed effects			
Parameters	Units	Estimates	R.S.E (%)
F_SDZ	%	99	<0.1
F_SMX	%	99	<0.1
F_TMP	%	99	<0.1
ka_SDZ	h^−1^	0.71	20.10
ka_SMX	h^−1^	0.59	16.40
ka_TMP	h^−1^	0.59	11.30
Vd_SDZ	L/kg	0.51	7.81
Vd_SMX	L/kg	0.62	10.10
Vd_TMP	L/kg	3.14	5.42
CL_SDZ	L/h/kg	0.15	6.00
CL_SMX	L/h/kg	0.15	8.72
CL_TMP	L/h/kg	1.53	4.77
β_Vd_SDZ_BW	–	0.65	40.70
β_Vd_SMX_BW	–	0.36	46.40
β_CL_SDZ_BW	–	0.42	41.40
β_CL_TMP_BW	–	0.42	23.10

Standard deviation of the random effect			
	Value	CV (%)	R.S.E (%)
ω_ka_SDZ	0.93	116.87	19.40
ω_ka_SMX	0.63	70.47	16.10
ω_ka_TMP	0.65	72.05	14.20
ω_Vd_SDZ	0.3	30.27	30.80
ω_Vd_SMX	0.24	24.31	20.50
ω_Vd_TMP	0.28	28.67	13.10
ω_CL_SDZ	0.25	25.76	19.11
ω_CL_SMX	0.23	22.82	19.30
ω_CL_TMP	0.27	27.48	11.90

Error model parameters			
b_SDZ	0.33		5.53
b_SMX	0.36		5.56
b_TMP	0.37		4.36

Abbreviations: b: proportional residual error; BW: bodyweight; Cl: clearance; CV: coefficient of variation; F: bioavailability; ka: absorption rate constant; R.S.E: relative standard error; SDZ: sulfadiazine; SMX: sulfamethoxazole; TMP: trimethoprim; Vd: volume of distribution; β_xx_SDZ_BW and β_xx_SMX_BW: covariate effect of “BW” on parameter xx; ω: standard deviation of random effects.

The unexplained IIV variability is represented by the omega parameters and the effect of a covariate on a typical value is represented by the beta parameters.

**Table 2:** Values of secondary parameters (computed based on the final model) following a single IV and an oral administration of TMP/SDZ or TMP/SMX to broilers.

	Route of administration			
	IV		oral	
Parameters	Mean values	Range (min-max)	Mean values	Range (min-max)
T_1/2β_ SDZ (h)	2.00	1.40–3.20	2.00	1.30– 2.90
T_1/2β_ SMX (h)	2.80	1.90–4.20	2.80	2.10–3.90
T_1/2β_ TMP (h)	1.50	0.80–4.20	1.50	0.90–3.80
AUC_∞_ SDZ (h × µg/mL)	187.00 ± 51.70	133.60–310.20	183.70 ± 59.40	97.20–292.30
AUC_∞_ SMX (h × µg/mL)	204.00 ± 40.10	153.60–310.60	253.30 ± 67.60	154.50–436.00
AUC_∞_ TMP (h × µg/mL)	4.00 ± 1.20	2.00–5.90	4.52 ± 1.80	2.00–10.10

Abbreviations: AUC_∞_: area under the curve from time 0 to infinity; IV: intravenous; SDZ: sulfadiazine; SMX: sulfamethoxazole; T_1/2β_: elimination half-life; TMP: trimethoprim.

Values are mean values of all individuals ± standard deviation.

**Figure 3:**
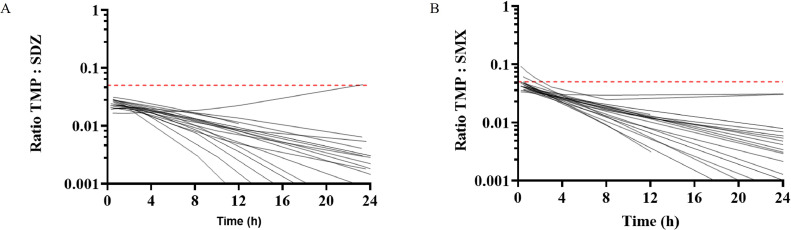
Scatter plot of the ratios evolution of (A) TMP/SDZ and (B) TMP/SMX over 24 h for each broiler following an oral administration. Each dot represents the total plasma concentration of TMP over the total plasma concentration of S obtained for each individual fit. Data over 24 h and below a ratio of 0.001 (corresponding to a TMP:S ratio of 1:1000) were censored. The 1:19 plasma ratio is represented by the red dashed line.

The evolution of the ratios TMP:SDZ or TMP:SMX over 24 h after an oral administration was plotted for each broiler included in the study (Figure 3). The plasma ratio of total concentrations of TMP:S remained below 1:19 in broilers for the combination TMP/SDZ and hardly reached 1:19 (up until a ratio of 1:11 for 1 broiler) for the TMP/SMX combination.

### Simulations

**Figure 4:**
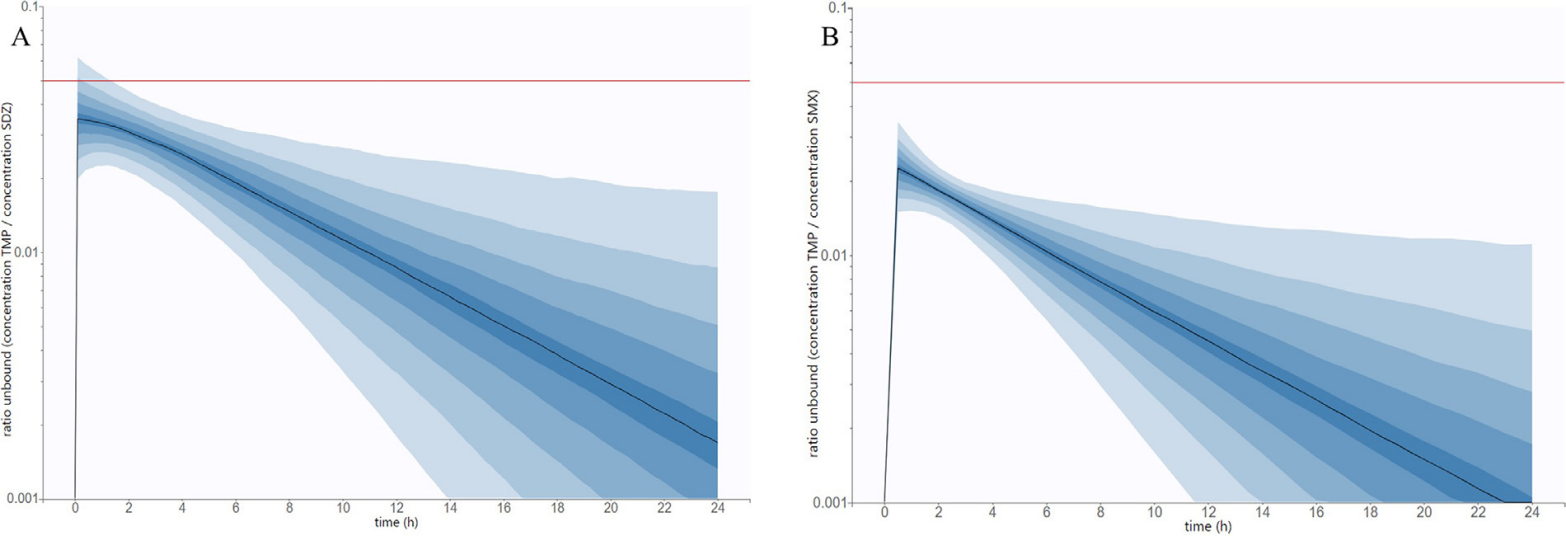
Simulation of the ratio of free concentrations of (A) TMP/SDZ and (B) TMP/SMX over 24h after a single oral administration. The median (n = 1,000 broilers) is represented by a solid black line and the 10% to 90% prediction interval divided into 9 blue areas, each one representing 10%. The 1:19 ratio is represented with a solid red line.

Based on the final models, the ratio of unbound concentration of TMP/SDZ and TMP/SMX over 24 h after oral administration at the recommended dosages were simulated for a population of 1,000 broilers that would have the same PK parameters (mean and variability) as broilers in our study (Figure 4). The plasma ratio of unbound concentration varies greatly over time for both combinations and only a very small fraction of individuals (∼5%) would reach the targeted ratio of 1:19 for a very limited time (<2 h after administration).

## DISCUSSION

The aim of this study was to evaluate the pharmacokinetics of 2 combinations of TMP/S which are frequently used in poultry farming, namely TMP/SDZ and TMP/SMX, using a population PK modeling approach and to compare the obtained *in vivo* TMP:S ratio with the 1:19 ratio, which is the standard used for susceptibility testing to TMP/S via MIC determination. To the best of our knowledge, this is the first study using population pharmacokinetics to investigate these TMP/S combinations in broilers.

During model development, the 1-compartment model gave the better fit, as outlined by the satisfying pcVPCs (see Figure 2) and by the other GOF plots (see Supplementary Figure 1). Moreover, results from the external validation for TMP/SDZ showed a good predictive ability of our model with previous PK studies, despite the different experimental conditions regarding the ages/weights of animals, the formulations of the drugs and the doses used. No external validation of the SMX model was done because of major differences of breed (laying hens versus broiler in our study) with physiology that could affect the PK of drugs (Queralt and Castells, 1985; Petritz et al., 2023). Overall, a very high confidence is obtained regarding the popPK models developed in the present study for prediction at the population level of broilers.

In this study, the population clearance of TMP (1.53 L/h/kg) was found to be 10 times higher than the population clearance of SDZ and SMX (0.15 L/h/kg for both sulfonamides). The same observation was obtained by Baert et al. (2003) and Petritz et al. (2023) with, however, lower values of SDZ, SMX and TMP clearances (Baert et al., 2003; Petritz et al., 2023). Thanks to the population PK analysis, the bodyweight of broilers was found to have a significant positive influence on the clearance of SDZ and TMP.

The typical values of the volume of distribution for SMX and SDZ were estimated at 0.62 L/kg and 0.51 L/kg, respectively. Overall, this is consistent with the literature as a value of 0.69 L/kg (following an IV administration) was reported for SMX (Queralt and Castells, 1985). Petritz et al. (2023) found a lower Vd for SMX (0.2 L/kg) but their study involved 3-yr-old laying hens compared to this study which involved 3 to 4-wk broilers (Petritz et al., 2023). Löscher et al. (1990) found a higher volume of distribution for SDZ (0.96 L/kg). The bodyweight was found to have a positive influence on the Vd of both sulfonamides. Trimethoprim was found to have a Vd about 5 to 6 times higher than both sulfonamides (3.14 L/kg) linked to a more extensive plasma to tissue distribution. This could mainly result from the chemical properties of both molecules as TMP is a lipid-soluble organic weak base whereas sulfonamides are weak organic acids (Garwacki et al., 1996).

Regarding the elimination half-life, TMP was found to have the lower average value (1.49 h) followed by SDZ (2.01 h) and SMX (2.83 h). In the current study, the absolute bioavailability after oral administration was very close to 100% for SMX, SDZ and TMP.

For TMP/S combinations, it is considered that TMP is 20 times more active than sulfonamides settling the current use of the TMP:S ratio of 1:19 for MIC determination and susceptibility reporting (EUCAST, 2017). Interestingly, as previously shown by Baert et al. (2003) for TMP/SDZ, the plasma ratio of 1:19 was not reached in any broilers for the combination TMP/SDZ and was observed only transiently for 2 broilers (up until a ratio of 1:11 for 1 broiler) for the combination TMP/SMX (see Figure 3). To propose a more accurate overview of TMP:S ratios than those previously reported by Baert et al. (2003), we only considered the unbound fractions (i.e. the fractions which are the only effective ones) and used the pop PK model to assess the ratios achieved in a large simulated population of broilers.

Both SDZ and TMP are known to bind highly to plasmatic proteins as their protein binding was reported in broilers to be 80% and 77%, respectively, in the summary of product characteristics of Adjusol (Summary of product characteristics of Adjusol, 2024). The protein binding for SMX was not found in the literature for broilers and was fixed at 62% in these simulations, which corresponds to values found *in vivo* for cows (Nielsen and Rasmussen, 1977) and sheep (De Backer et al., 1981). Following oral administration of the combination TMP/SDZ or TMP/SMX at the recommended doses and considering the respective unbound fraction for each molecule, the synergistic plasma ratio concentrations of 1:19 were not reached for the combination TMP/SMX. It was transiently observed during 1.5 h for animals between the 90th and 95th percentiles (only 5%) of the simulated broilers for the combination TMP/SDZ (see Figure 4). Simulations showed that the TMP:S ratios are not constant over time and vary greatly, as TMP/SDZ ratios ranging from about 1:40 to 1:550 would be reached within the 5-95th percentiles 12 h after oral administration. Ratios of TMP/SMX are even lower for the same timepoint, as ratios from about 1:70 to 1:1,000 would be observed within the same prediction interval.

These simulations highlighted that the dose ratio of 1:5 which is currently used for the TMP/S combinations in the treatment of broilers is most likely sub optimal (as similarly observed in other farm species, including pigs [Viel. et al., 2023]) given that the target ratio should be 1:19. Such findings may give rise to concerns in the field regarding the relevance of susceptibility testing with 1:19 ratio for many veterinary bacterial strains. The most synergistic *in vitro* ratio TMP:S actually varies greatly as it corresponds to the ratio of the MIC of each molecule used alone, meaning there is no universal “optimal” ratio (Barnett and Bushby, 1970; Bushby, 1980).

Indeed, preliminary studies from our team showed that the highest synergistic effect of TMP/S on *Escherichia coli* can be obtained with a large range of ratios depending on the strain and the sulfonamide (from 1:32 to 1:320 for SMX and from 1:16 to 1:1,280 for SDZ) (Boulanger et al., 2023). Due to the rather low TMP:S ratios predicted by our model for most of broilers after treatment with licensed doses, pharmacodynamic (**PD**) studies with pathogenic bacterial species commonly found in broilers are needed to better understand the effects of each drug alone and in combination. Our team is currently investigating this thanks to a mechanistic PD modeling approach, which will eventually help to determine whether the in vivo TMP:S ratios could be effective or whether TMP/S dosing regimens should be revised and optimized (Wicha et al., 2017).

Lastly, our study has some limitations. One of these concerns the use of gavage for oral administration, which is not representative of the current farming practice where broilers are usually treated through drinking water and which could increase the inter-individual variability regarding exposure (Ferran et al., 2020; Chassan et al., 2021). However, this was the easiest way to control the administered doses of each drug and thus have a reliable estimation of the bioavailability. Also, the unbound fraction value for SMX used in the simulations may have been biased as the true value in broilers may be different to that in cows and sheep.

To conclude, combining both the current PK and future PD data and optimizing the actual dosage regimens of TMP/S in this species through PK/PD modeling will be necessary to preserve the efficacy of this important antimicrobial combination in veterinary medicine.

## DISCLOSURES

The authors declare no conflicts of interest.

## Appendix Supplementary materials

Image, application 1

## Footnotes

Supplementary material associated with this article can be found in the online version at doi:10.1016/j.psj.2024.104200.
