# Supplementary material for: Pharmacokinetic modeling of sulfamethoxazole-trimethoprim and sulfadiazine-trimethoprim combinations in broilers
Source: Poult Sci. 2024 Aug 8;103(11):104200. doi: 10.1016/j.psj.2024.104200 (PMC11399637; doi:10.1016/j.psj.2024.104200)
Supplement: Supplementary file 1 [file mmc1.docx]

**Supplementary data**


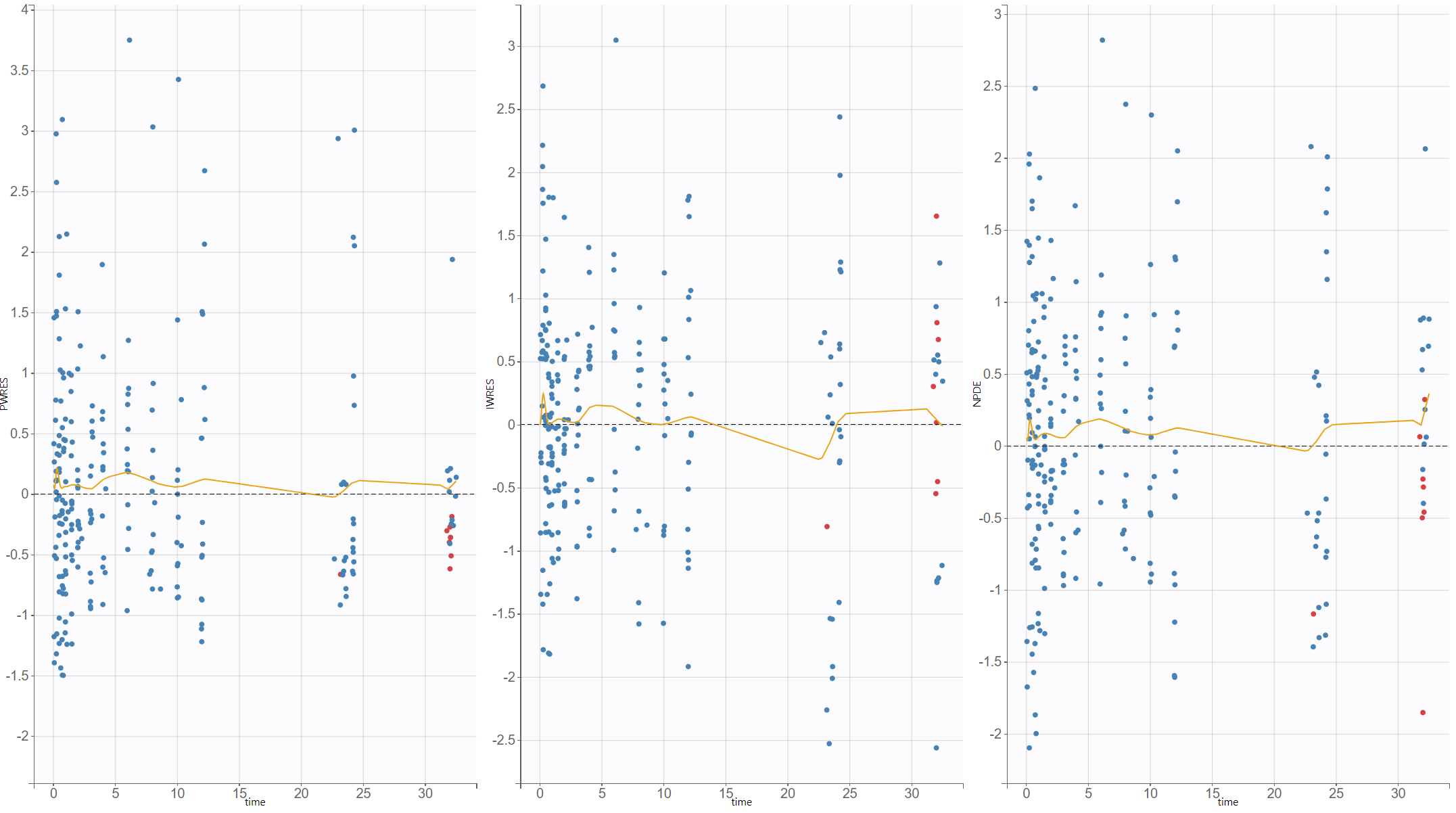


B


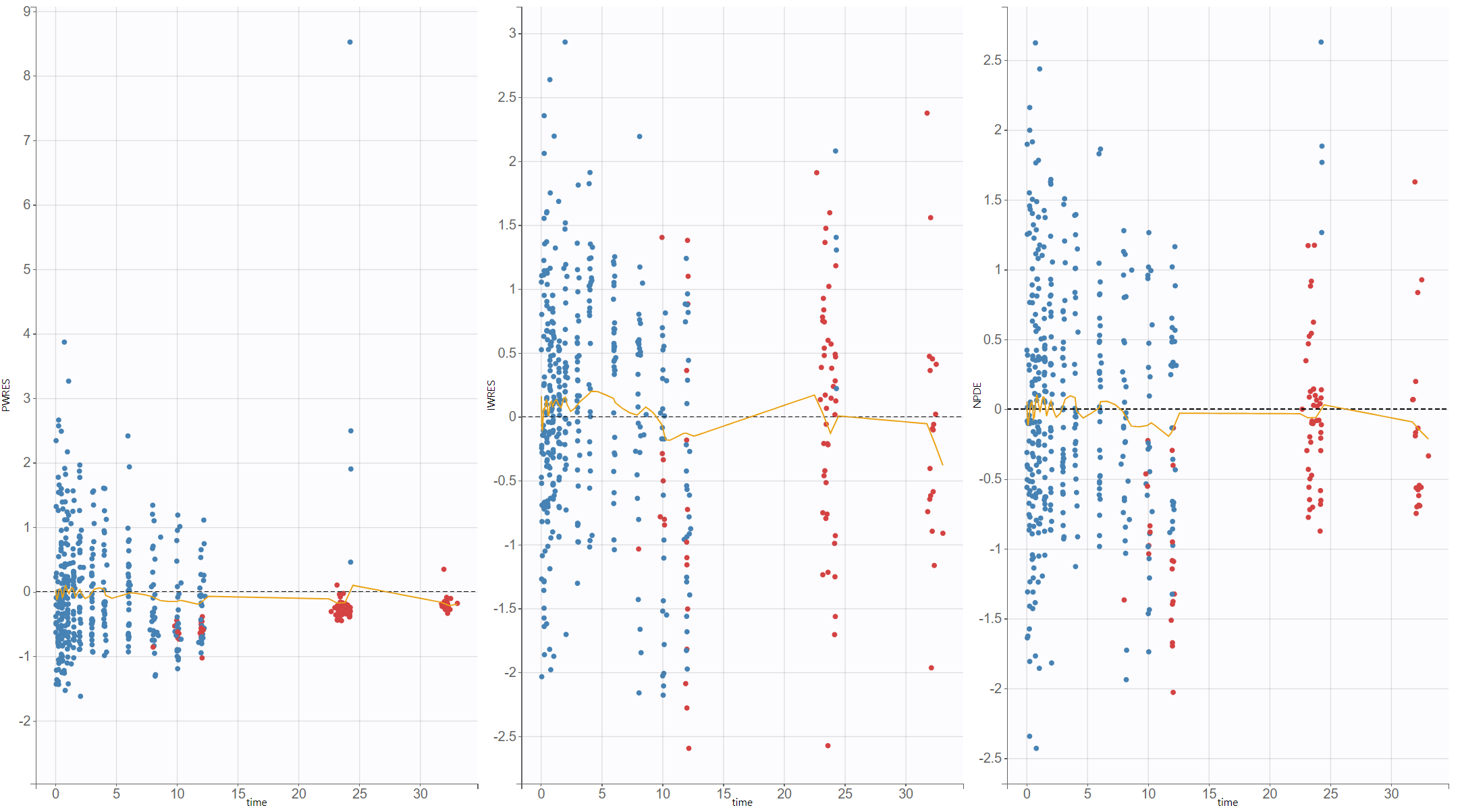


C


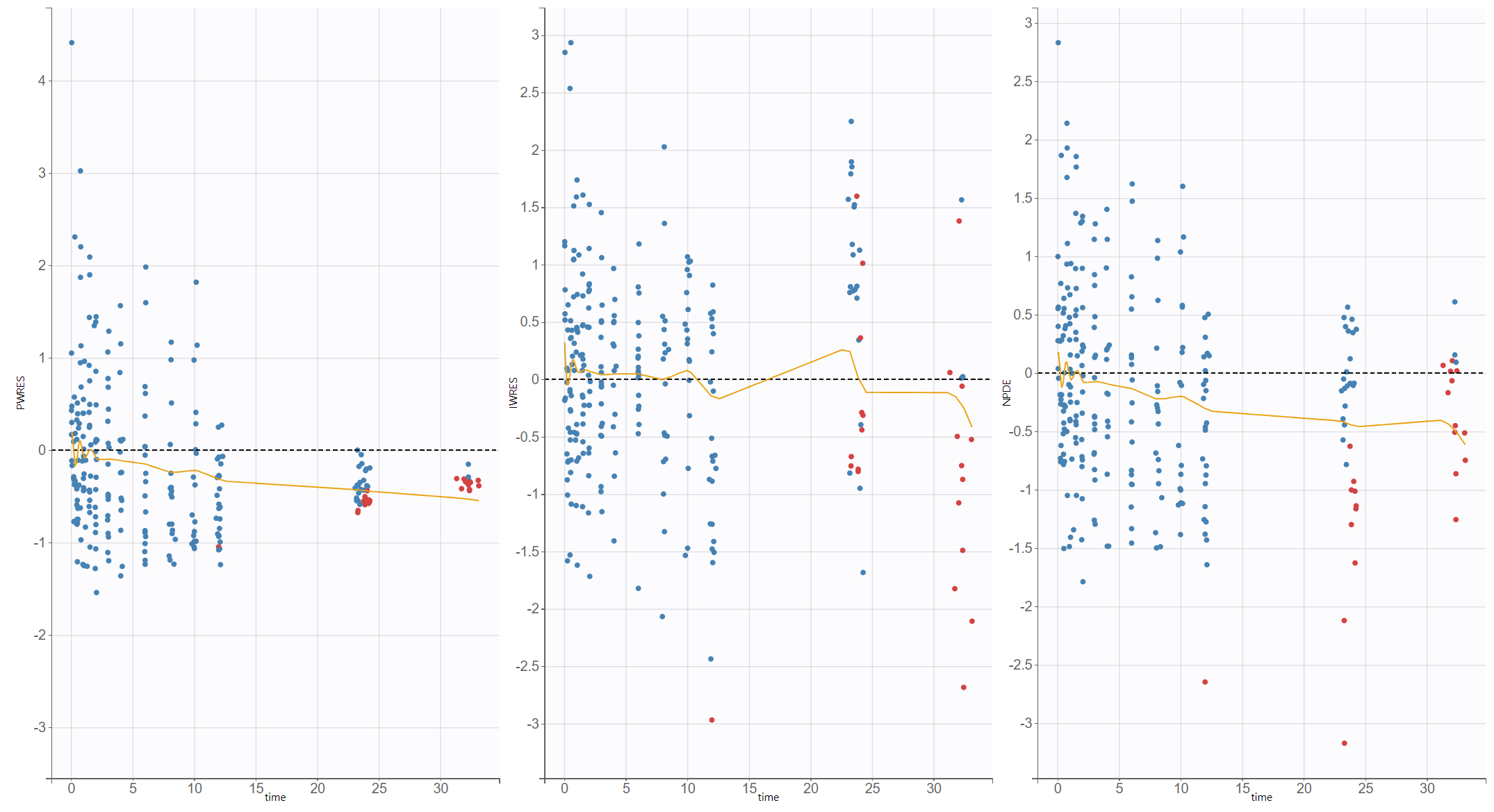


A

Supplementary data Figure 1: Graphical evaluation of PWRES (left), IWRES (in the middle) and NPDE (right) of the (A) SDZ, (B) SMX and (C) TMP final models. Data are represented in blue dots, censured data are represented in red dots and the spline interpolatio n is represented with a yellow line.


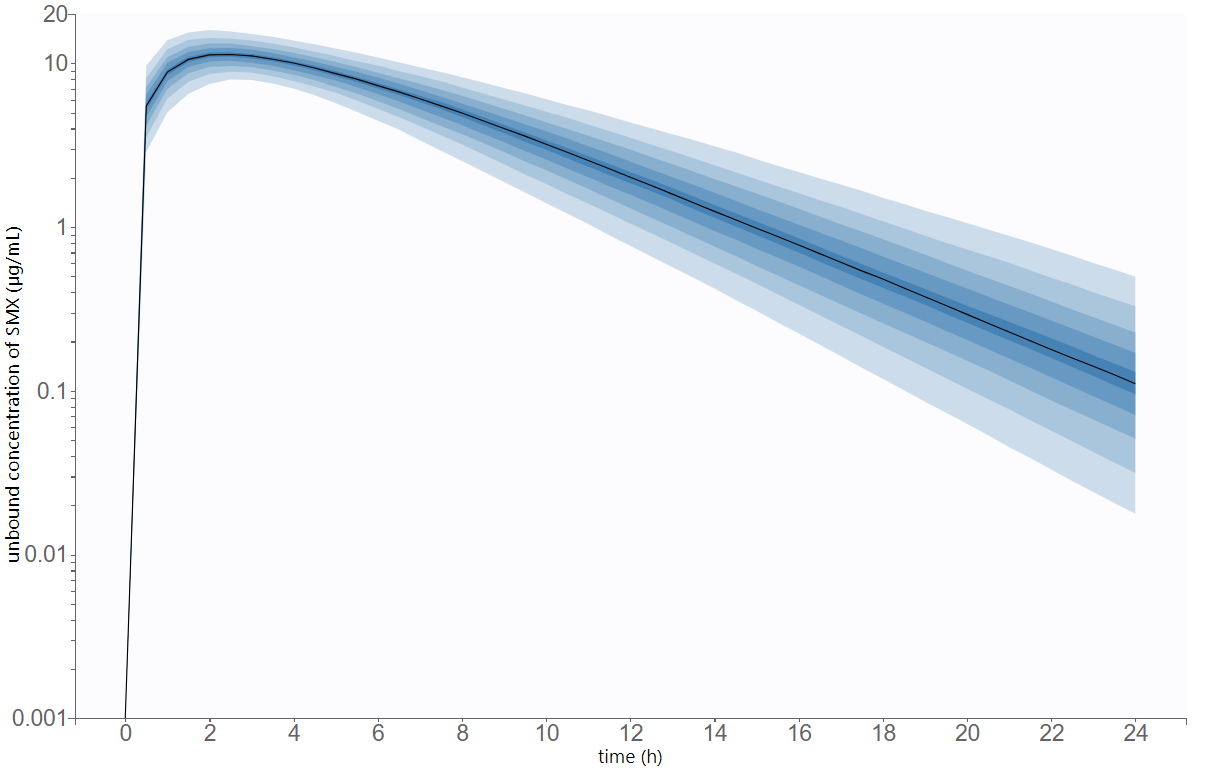

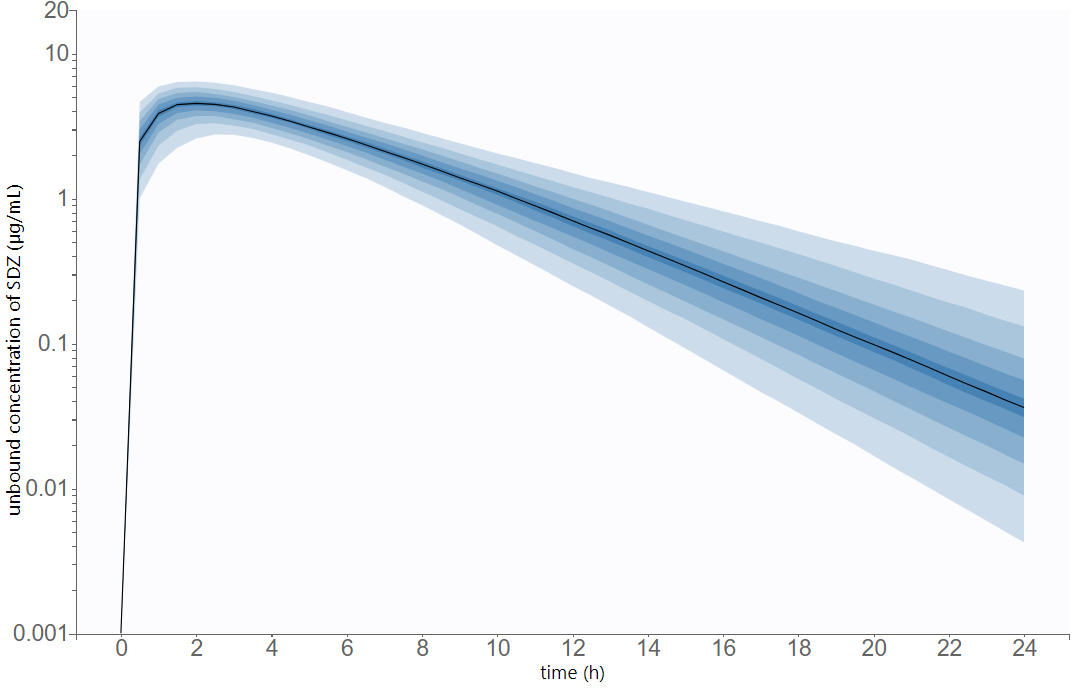

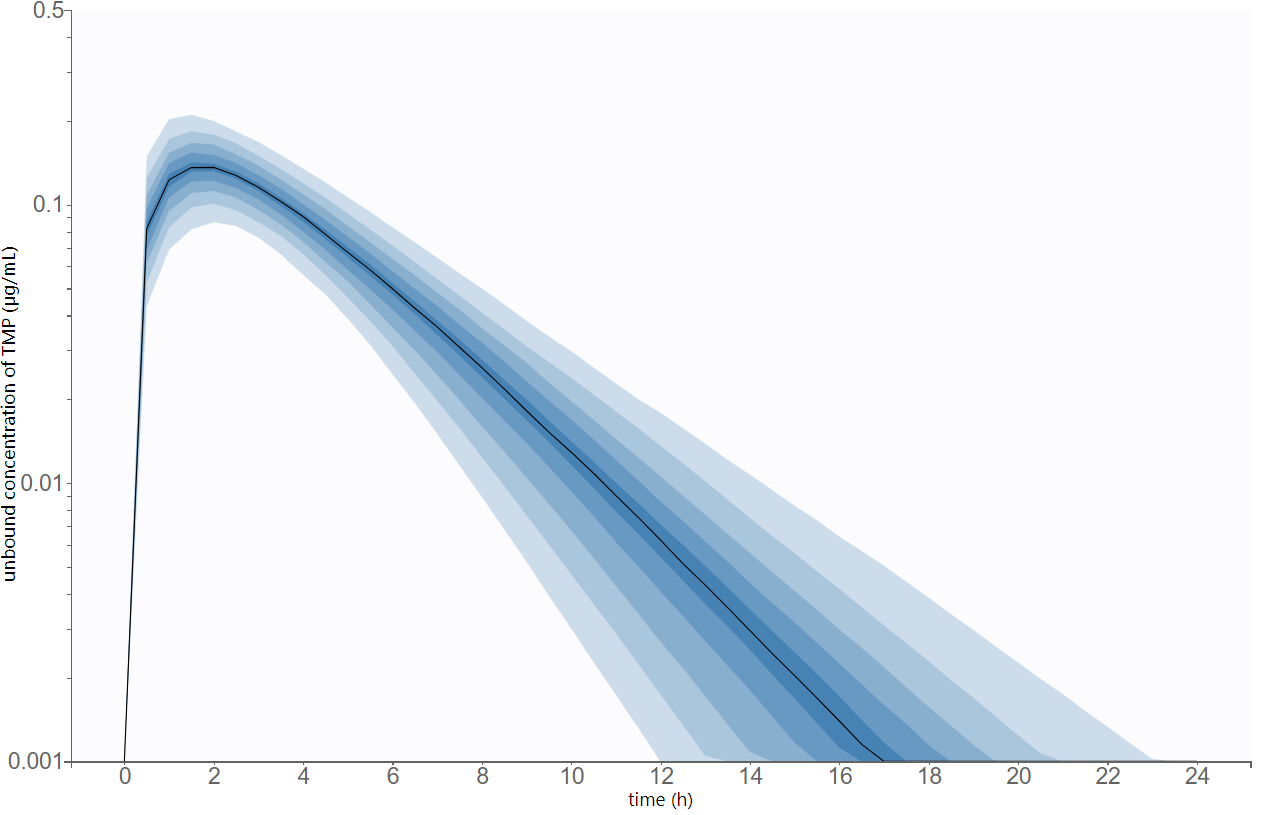

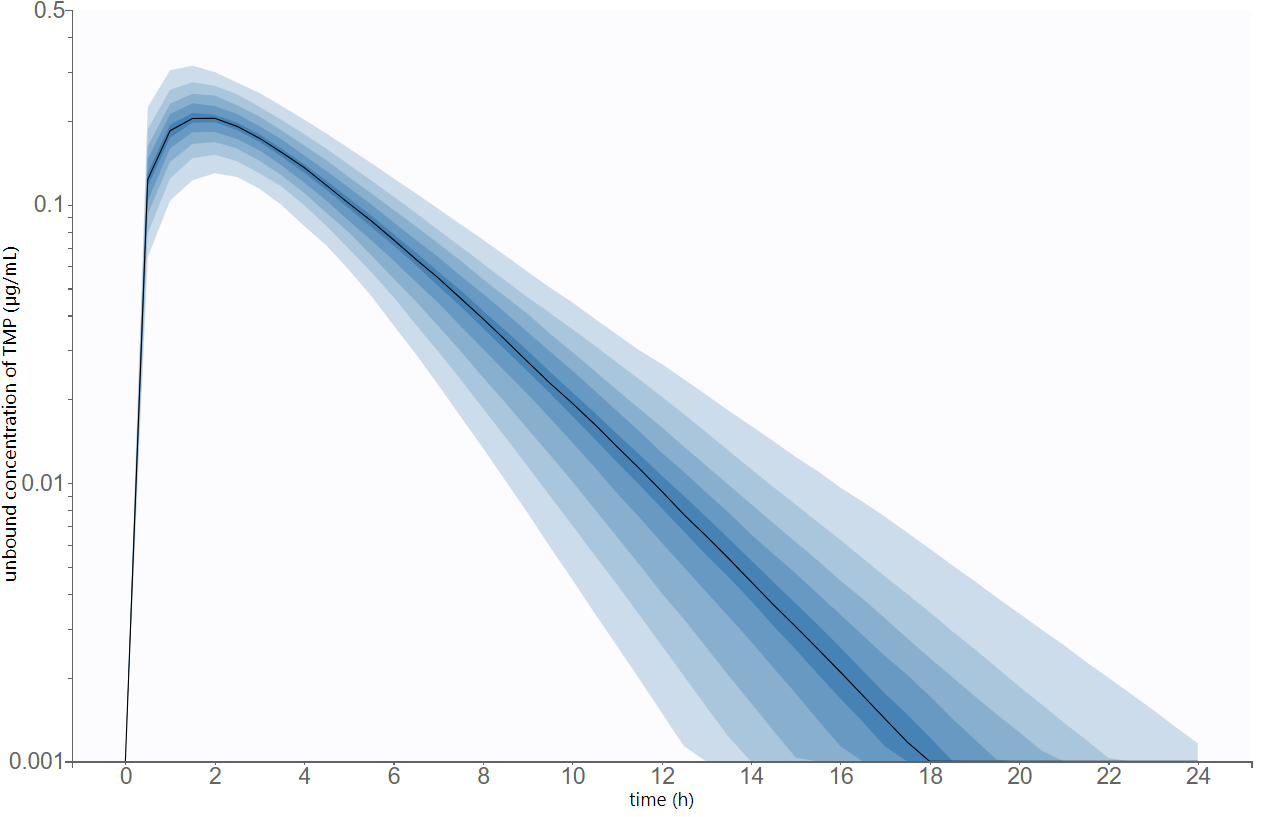


C

A

B

D

**$**

Supplementary data Figure 2: Simulation of the free concentration following the oral administration of (A) SDZ (25 mg/kg), (B) SMX (37.5 mg/kg), (C) TMP (5 mg/kg) and (D) TMP (7.5 mg/kg) over 24h. The median (n = 1000 broilers) is represented with a solid black line and the 10%-90% prediction interval divided into 9 blue areas, each one representing 10%.


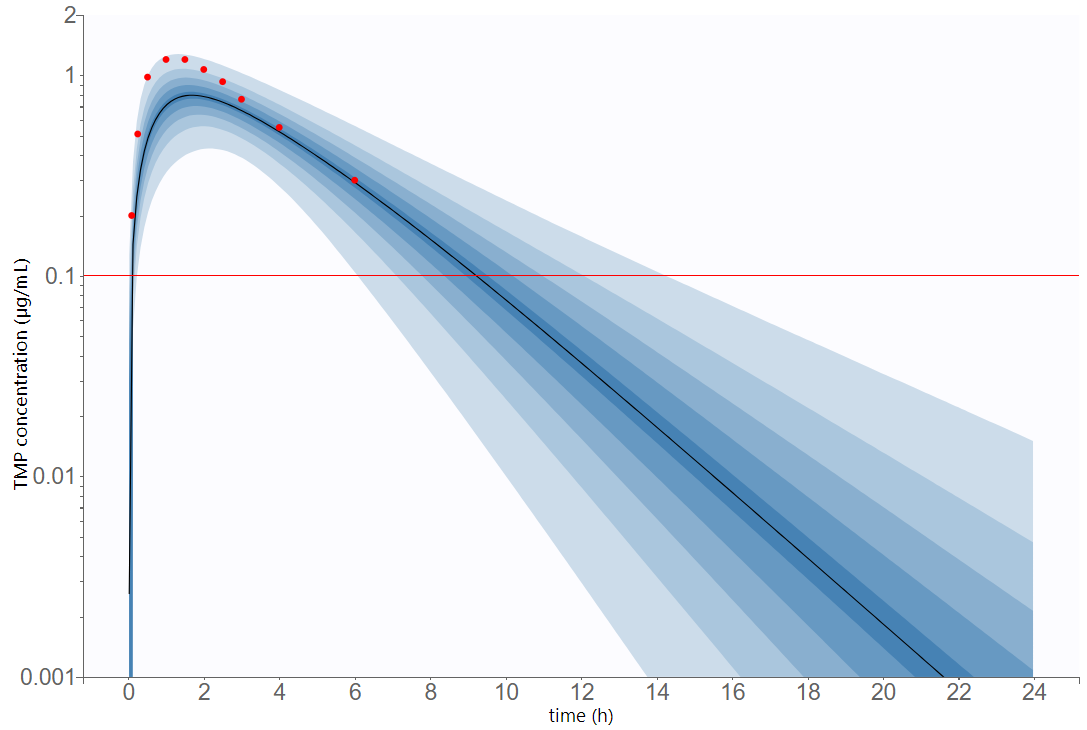

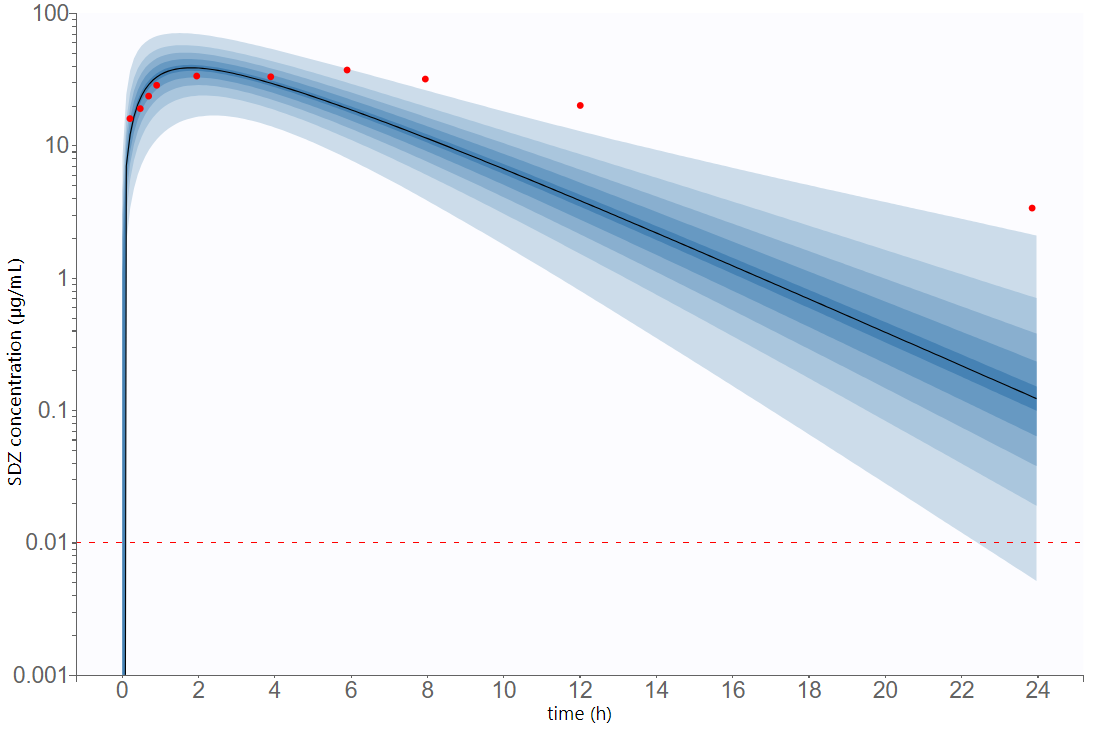

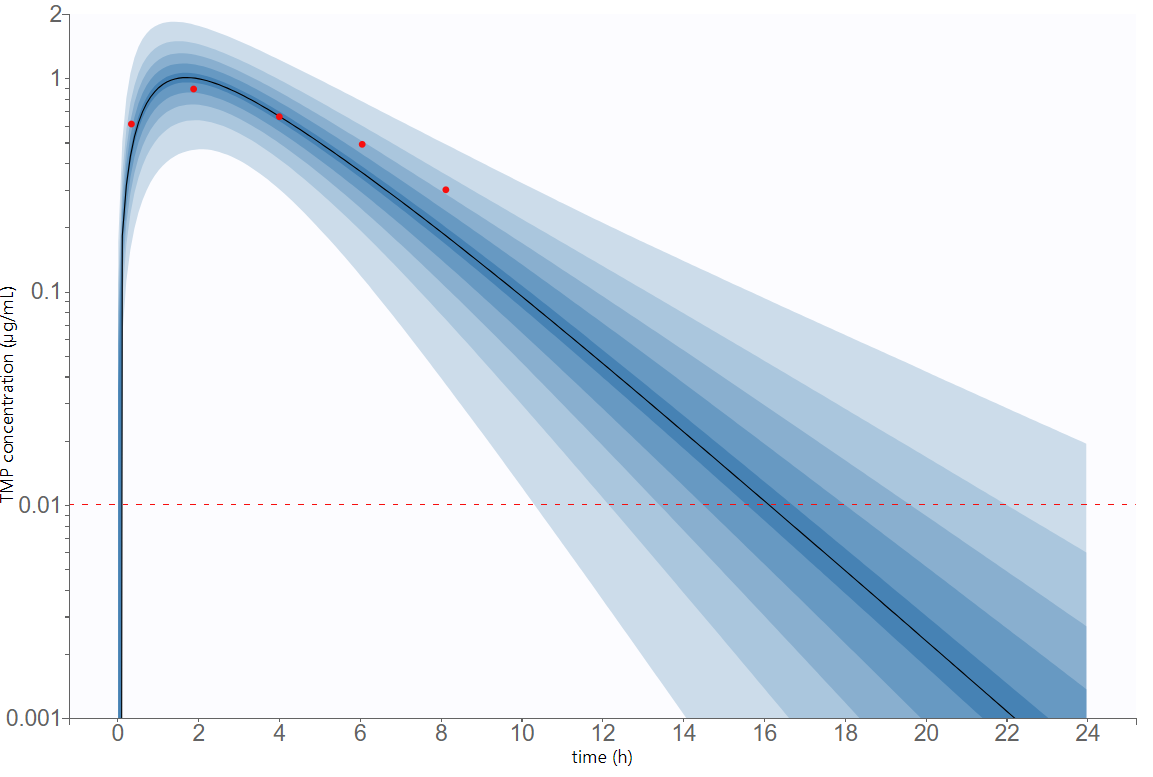

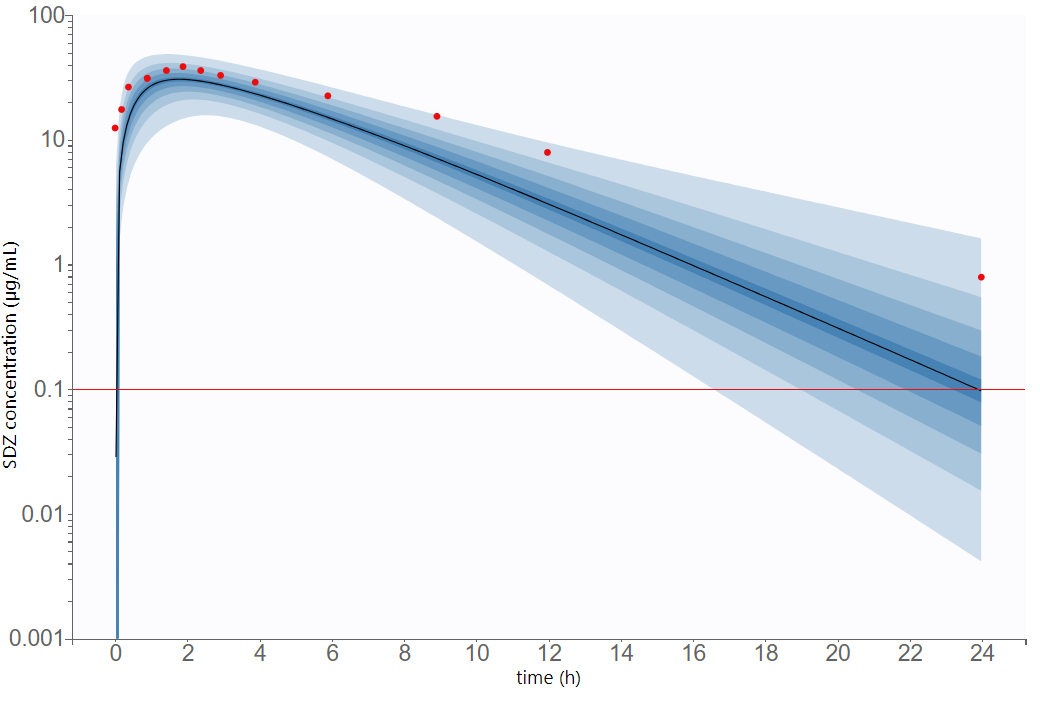


D

A

B

C

Supplementary data Figure 3: External validation of the final (A-B) SDZ and (C-D) TMP model using Baert et al. (left panel) and Löscher et al. (right panel) studies. The median (n=1000 broilers) is represented with a solid black line and the 5%-95% prediction interval is divided into 9 blue areas, each one representing 5%. Mean data from respective studies are plotted in red dots. The limit of quantification (LOQ) in Baert et al. Study (0.1µg/mL for both SDZ and TMP) is represented with a solid red line. Only the limit of detection (LOD) was described in the Löscher et al. study (about 10 ng/mL for both SDZ and TMP) and is represented with a dashed red line.

Supplementary Table 1: Values of the correlations between random-effects estimated thanks to a full variance-covariance matrix. Values ≤ 0.2 were removed for the simulations.

| **Correlations** | **Values** | **R.S.E (%)** |
| --- | --- | --- |
| CL_SMX - CL_SDZ | 0.58 | 165 |
| CL_TMP – CL_SDZ | 0.71 | - |
| CL_TMP – CL_SMX | 0.55 | 62.4 |
| Vd_SDZ – CL_SDZ | 0.14 | 202 |
| Vd_SDZ – CL_SMX | 0.3 | - |
| Vd_SDZ – CL_TMP | -0.19 | - |
| Vd_SMX – CL_SDZ | 0.74 | 127 |
| Vd_SMX – CL_SMX | 0.69 | 26.9 |
| Vd_SMX – CL_ TMP | 0.55 | 74.9 |
| Vd_SMX – Vd_SDZ | 0.43 | 345 |
| Vd_TMP – CL_SDZ | 0.6 | - |
| Vd_TMP – CL_SMX | 0.47 | 40.8 |
| Vd_TMP – CL_TMP | 0.22 | - |
| Vd_TMP – Vd_SDZ | 0.6 | - |
| Vd_TMP – Vd_SMX | 0.75 | - |
| ka_SDZ – CL_SDZ | -0.11 | 273 |
| ka_SDZ – CL_SMX | 0.23 | 295 |
| ka_SDZ – CL_TMP | -0.2 | - |
| ka_SDZ – Vd_SDZ | 0.75 | - |
| ka_SDZ – Vd_SMX | 0.2 | 410 |
| ka_SDZ – Vd_TMP | 0.17 | - |
| ka_SMX – CL_SDZ | 0.36 | - |
| ka_SMX – CL_SMX | 0.56 | 30.0 |
| ka_SMX – CL_TMP | 0.27 | - |
| ka_SMX – Vd_SDZ | 0.56 | 134 |
| ka_SMX – Vd_SMX | 0.55 | - |
| ka_SMX – Vd_TMP | 0.32 | - |
| ka_SMX – ka_SDZ | 0.74 | - |
| ka_TMP – CL_SDZ | 0.11 | - |
| ka_TMP – CL_SMX | 0.29 | 69.8 |
| ka_TMP – CL_TMP | -0.26 | - |
| ka_TMP – Vd_SDZ | 0.81 | 60.2 |
| ka_TMP – Vd_SMX | 0.39 | - |
| ka_TMP – Vd_TMP | 0.51 | - |
| ka_TMP – ka_SDZ | 0.82 | - |
| ka_TMP – ka_SMX | 0.72 | - |

Abbreviations: CL = clearance; Vd = volume of distribution ; ka = absorption rate constant; SMX = sulfamethoxazole; SDZ = sulfadiazine; TMP = trimethoprim; RSE = relative standard error
